# S2, S3, and S4 Sacral Dermatomal Evoked Potentials: Technical Parameters and Normative Values

**DOI:** 10.1097/WNP.0000000000001206

**Published:** 2025-09-17

**Authors:** Prasad Malladi, Llwyd Orton, Sara Simeoni, Jalesh Panicker

**Affiliations:** * UCL Institute of Neurology—Brain Repair and Rehabilitation, London, United Kingdom;; † Department of Uro-Neurology, The National Hospital for Neurology and Neurosurgery, University College London Hospitals, London, United Kingdom; and; ‡ Department of Life Sciences, School of Healthcare Science, Manchester Metropolitan University, Manchester, United Kingdom.

**Keywords:** Sacral dermatomal evoked potentials, Pudendal SEP, Tibial SEP, Normative values, Cauda equina syndrome, Tarlov cysts

## Abstract

**Purpose:**

The existing literature on the sacral dermatomal evoked potentials (dSEPs) is limited. This study aims to develop stimulating parameters and establish normative values for S2, S3, and S4 sacral dermatomes in healthy adult populations.

**Methods:**

Twenty healthy adult volunteers were enrolled in the study. The study was ethically approved, and written consent for participation was provided. All participants underwent tibial, pudendal, S2, S3, and S4 dSEPs. Stimulating and recorded parameters were established for all evoked potentials. P40 latency, amplitude, and interpeak parameters were calculated for each waveform. A comparison was made between tibial, pudendal somatosensory evoked potentials, and all sacral dSEPs. Normative values were generated for sacral dSEPs based on various height, age, and Body Mass Index (BMI) parameters.

**Results:**

The sacral dSEPs were well tolerated and recorded in all participants. S2 latency was mildly influenced by age and height, while S3 and S4 latencies were unaffected by age or height. BMI does not affect the S2 and S3 latencies but mildly affects the S4 latency. Sacral dSEP latencies were comparable with pudendal SEPS but not with the tibial somatosensory evoked potential.

**Conclusions:**

The S2, S3, and S4 sacral dSEPs can be used as diagnostic tools to evaluate sacral nerve lesions such as cauda equina syndrome and Tarlov cysts, complementary to pudendal and tibial somatosensory evoked potentials.

## Abstract 2

Supplemental Digital Content is Available in the Text.

Short latency somatosensory evoked potentials (SEPs) have been in clinical use for more than five decades to evaluate predominantly the central nervous system.^1^ The tibial and median SEPs are mixed EPs generated by activating sensory fibers, such as tactile, vibration, and sensory afferents to muscle spindles and Golgi tendon organs through multiple dorsal roots. Segmental SEPs, such as sural or saphenous nerve SEPs, result from the activation of large and small diameter myelinated sensory fibers and a small amount of Aδ fibers that travel through the adjacent two dorsal roots.^2,3^

Dermatomal somatosensory evoked potential (dSEP) is recorded by stimulating the cutaneous nerve fibers in each dermatome's autonomous nerve zone. In dSEPs, the cutaneous afferent signals ascend through a single dorsal nerve root, enter the spinal cord, and pass through the dorsal column-medial lemniscus system, similar to SEPs. Tibial SEP and pudendal SEP are good at evaluating the sensory pathways of the central nervous system, but they lack the specificity to assess individual sacral roots' sensory functions.^4–6^ Because dSEPs pass through a single dorsal root, they are helpful in assessing sacral dorsal nerve roots selectively in conditions such as sacral Tarlov cysts, cauda equina lesions, or root compression due to tumors.^7–11^ Hence, there is a clinical need to develop S2, S3, and S4 dSEPs to evaluate individual sacral sensory root functions. Normative values for cervical C4 root to sacral root S1 dSEPs are available in the literature.^6,12–15^ These studies have significant variations in technical parameters, such as the type of electrodes used for stimulation and recording. In addition, many of the dSEP normative studies did not consider factors such as height, age, or BMI. To our knowledge, normative values for S2, S3, or S4 dSEPs have never been established. Furthermore, simultaneous comparisons between sacral dSEPs and tibial and pudendal SEPs are lacking. Therefore, this study aimed to generate normative values for S2, S3, and S4 dSEPs, investigate the relationship between tibial, pudendal SEPs, and sacral dSEPs and provide normative values for clinical use.

## METHODS

### Subjects

Eleven healthy volunteers and nine individuals with non-neurogenic voiding dysfunction consented to participate in this study. The mean height for men was 176 (168–190) + 8.1 cm, and that for women was 162 (150–183) + 8.8 cm. Similarly, the mean age for men was 35 (26–49) + 10.6, and for women it was 39 (20–75) + 16.6. The mean BMI for men was 24.5 ([19.3–31.9] + 5.5 kg/m^2^) and that for women was 25.9 ([17.7–39.9] + 6.6 kg/m^2^). This study was approved by the North East-Tyne and Wear South Research Ethics Committee (REC reference: 21/NE/0194). A neurologist conducted a clinical examination of the volunteers who provided written consent to determine their eligibility for participation. None of the volunteers had a history of signs and symptoms indicating sensory or motor disorders. Volunteers were compensated in accordance with the ethics committee recommendations for their participation in this study. Tibial SEP, pudendal SEP, and S2, S3, and S4 dSEPs were recorded in all 20 subjects. Participants underwent tibial and pudendal SEPs while lying supine, and for dSEPs, they lay on their left side. All participants were awake but kept their eyes closed throughout the test, and the room temperature was maintained at 22°C.

### Stimulus Parameters

**FIG. 1.**
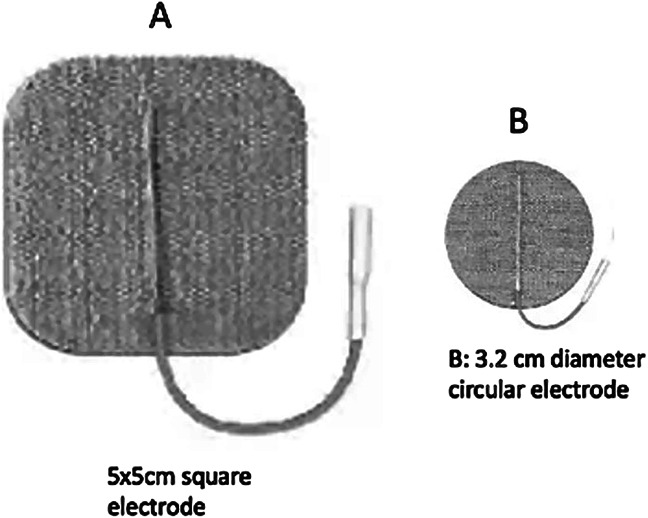
Multilayer hydrogel stimulating electrodes. **A,** Square electrodes were used for S2 and S3 dSEPs. **B,** Circular electrodes were used for S4 dSEPs. dSEPs, dermatomal somatosensory evoked potentials.

The electrode size and placement of stimulating electrodes are vital to recording consistent and large amplitude dSEPs. Various electrodes, such as ring electrodes,^16^ bar electrodes,^2,17^ Ag–AgCl electrodes,^18^ and small-size surface gel electrodes,^13,19^ were tested in dSEPs. We tested all these stimulating electrodes and were most satisfied with the advanced multilayer hydrogel stimulating electrodes, as shown in Fig. 1. These advanced electrodes evenly distribute the stimulating current over the surface of the electrode and offer high conductivity, low skin-contact impedance, and high robustness for repeat uses.^20,21^ These electrodes produce higher cortical amplitudes with no difference in latency. In addition, none of our volunteers reported any pain or discomfort with these stimulating electrodes.

**FIG. 2.**
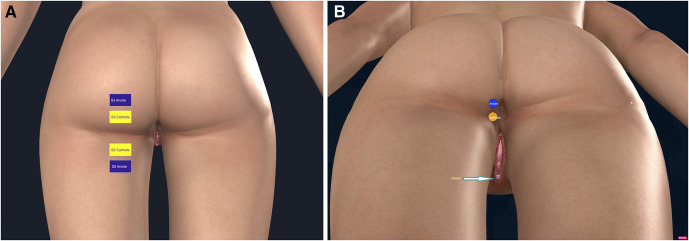
**A,** Electrode placements for S2 and S3 dSEPs. For S2 dSEP: Cathode was placed 2 cm distal to the gluteal fold. For S3 dSEP: Cathode was placed 2 cm proximal to the gluteal fold. Anode electrodes were always placed 2 cm away from the cathode. **B,** Electrode placements for S4 dSEPs. The electrodes were positioned adjacent to the anal orifice, immediately lateral to the mucocutaneous junction. Image source: Adapted and edited using 3D4 anatomy software (Anatomy, 2020) with permission from 3D4Medical. Adaptations are themselves works protected by copyright. So to publish this adaptation, authorization must be obtained both from the owner of the copyright in the original work and from the owner of copyright in the translation or adaptation. dSEPs, dermatomal somatosensory evoked potentials.

Applying the stimulating electrodes to the skin at a consistent place is essential to assess the reproducibility of any of the EP studies. Because identifying the gluteal fold is straightforward, a standard electrode placement technique is proposed in this study using the gluteal fold as a landmark. Foerster tactile dermatome map^22^ was used to determine the S2 autonomous zone. The S2 dermatome area is a wide strip extending from the popliteal fossa to the mid-gluteal area. For S2 dSEP, the cathode electrode was placed 2 cm distal to the gluteal fold on a virtual line connecting the gluteal fold's mid-point to the popliteal fossa's mid. The anode electrode was placed 2 cm distal to the cathode electrode, as shown in Fig. 2A. Foerster dermatomal map did not reveal the exact distribution of the S3 and S4 dermatomes. Hence, the electrode placement for the S3 dermatome was chosen based on the International Standards for Neurological Classification of Spinal Cord Injury Scale-2019^23^ and the distribution of the perineal branch of the posterior femoral cutaneous nerve, consisting of S3 fibers.^24^ The cathode for the S3 dermatome was placed 2 cm proximal to the gluteal fold, as shown in Fig. 2A, and its anode electrode was placed 2 cm distal to it. The cutaneous area immediately lateral to the mucocutaneous junction is supplied by the hemorrhoidal branch of the pudendal nerve that has S4 sensory fibers.^25–27^ To avoid co-stimulation, small 3.2 cm circular electrodes were used in the S4 dSEP, placed ipsilaterally around the anal orifice and immediately lateral to the mucocutaneous junction, as shown in Fig. 2B. The sensory perception threshold was identified by gradually increasing the electrical stimulation until the subject reported a sensation. The simulation strength was aimed at 3 times the sensory perception threshold but lowered to 2.5 times the sensory perception threshold if any muscle twitching was noted under the electrodes. Electrical stimulations were given with a constant current square wave pulse of 0.2 milliseconds duration, delivered at 3.1 Hz.

### Recording Parameters

American Clinical Neurophysiology Society (ACNS) guidelines^28^ were followed throughout this study while recording or reporting the EPs. Single Cadwell Sierra Summit EMG/EP equipment was used throughout the study. High- and low-frequency filters of 1 Hz and 3 kHz were used.^29,30^ One hundred milliseconds of analysis time was used with 10 milliseconds/division screen duration, with sensitivity at 2 μV/division. A three-channel montage C3′-Fz, Cz′-Fz, and C4′-Fz was used per ACNS guidelines. Even though the Cz′-Fz central channel was used to measure latency and amplitude, additional C3′-Fz and C4′-Fz were used to identify the field of central peaks. These two additional channels help differentiate noise from the genuine response.^2^

## RESULTS

### Pain Tolerance

All subjects tolerated the dSEP studies well. They were asked to fill out a numerical pain rate scale between 0 and 10, where 0 means no pain and 10 is the worst possible pain^31^ because of electrical stimulations. All subjects scored no more than one after the test, as shown in **Supplemental Digital Content 1** (see **Table 1**, http://links.lww.com/JCNP/A355), suggesting they tolerated the test well.

### Tibial SEP

All sacral dSEP studies were done using stimulating pads. Hence, the tibial SEP study was also performed using stimulating pads. The tibial SEP data in this study were compared with published tibial SEP studies to assess any statistical differences because of the usage of stimulating pads. Ten published studies were selected with a similar sample size to this study, and their mean latencies were given in **Supplemental Digital Content 1** (see **Table 2**, http://links.lww.com/JCNP/A355). This study showed the tibial mean SEP latency of 42.4 milliseconds, 2.7 milliseconds more than the average mean latency of 39.7 milliseconds of published studies. A list of all tibial SEP studies was given in **Supplemental Digital Content 1** (see **Table 1**, http://links.lww.com/JCNP/A355). The tibial latency in this study is greater than any of the 10 published studies except for study number 9. The *P*-value of the Kolmogorov–Smirnov test (0.200), Shapiro–Wilk (0.555), Skewness (0.379), and Kurtosis (0.338) showed that the current tibial SEP latency data are normally distributed. One-sample *t*-test (*t*-value: 4.738, *P* < 0.001 for one-sided and two-sided) showed that the observed mean latency difference of 2.7 milliseconds is statistically significant.

### Stimulus Strength

Determining the optimum stimulus strength required for sacral dSEPs before recruiting healthy subjects is essential. The impact of stimulus strength on cortical latency was assessed on one healthy volunteer owing to the difficulty of maintaining volunteer cooperation for several hours. The electrical stimulation perception threshold (EPT) was defined as the volunteers' perception of the first sensation of electrical stimulation when the electrical stimulation was gradually increased by 0.5 mA in each incremental step. Two runs of 200 averages were recorded for each stimulus strength. The P1 latency and P1-N1 amplitude were measured using the grand average of two runs. The effects of increased stimulus strength on the S2, S3, and S4 dSEP cortical latencies and amplitudes are shown in **Supplemental Digital Content 1** (see **Figures 1 and 2**, http://links.lww.com/JCNP/A355). The S2, S3, and S4 dSEP latencies and amplitudes plateaued when the stimulus strength was three times the EPT. However, the amplitude or latency change between 2.5 and 3 times EPT was not clinically significant. Hence, a stimulus strength of 3 times the EPT was chosen for all dSEPs but lowered to 2.5 times the EPT if there was any muscle twitch under the stimulus electrodes. However, the upper limit of current strength was arbitrarily limited to 50 mA owing to the risk of stimulating adjacent dermatomes or the underlying muscle.

### Effective Size

The optimum sample size for this study was calculated using the effective size. The effective size was calculated by subtracting the current tibial SEP mean latency from the mean of 10 published studies given in **Supplemental Digital Content 1** (see **Table 2**, http://links.lww.com/JCNP/A355), divided by their mean SD.^32^ The calculation results showed that the optimum sample size required was 23 to get a power of 0.9 with an effective size of 0.71 for a *P*-value of 0.05. The current sample size is 20, with an effective size of 0.746, close to the optimum sample size of 23.

### Sacral dSEPs

The S2, S3, and S4 dSEPs were recorded from 20 healthy volunteers. Cortical latencies and amplitudes were measured after grand averaging 2 runs, each with 200 averages. Kolmogorov–Smirnov (*P* = 0.200), Shapiro–Wilk (*P* = 0.440), and Skewness (*P* = −0.986), and Kurtosis (*P* = −0.637) tests showed that the cortical latencies were normally distributed. The linear regression analysis of the S2 dSEP latency data showed that the subject's BMI does not affect the latency. However, the combination of height and age showed an 18.1% variance in the S2 dSEP latency. A linear regression equation using SPSS software (version 28.0.1.1) was derived for the S2 latency as 15.9 + (0.049) * (age) + (0.117) * (height). The regression analysis showed that the S3 dSEPs latencies are unaffected by an individual's age, height, or BMI. Similarly, the S4 dSEP data showed that the subject's BMI explains 13.4% of the variance in the S4 dSEP latency, and hence, the linear regression equation for the S4 dSEP latency was derived as 42.2 + (−0.187) * BMI. The S2, S3, and S4 dSEP amplitude data suggest that height, age, and BMI do not have any predictive value for sacral dermatomal amplitudes.

### Pudendal SEP

**FIG. 3.**
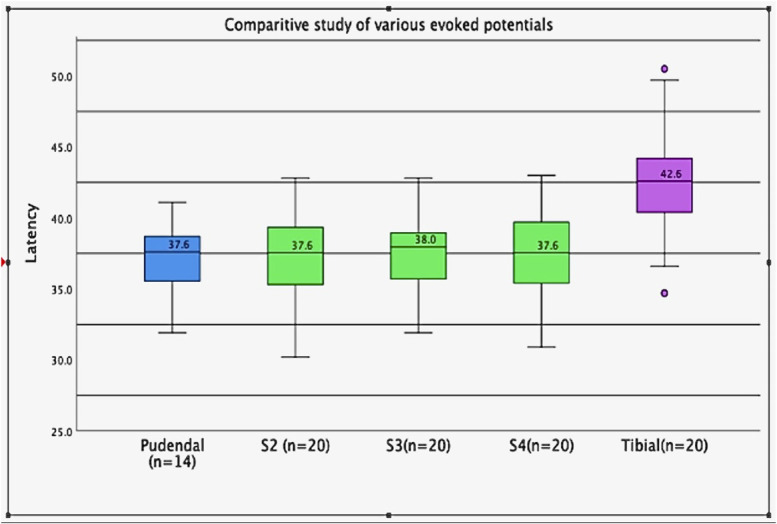
Comparison of S2, S3, and S4 dSEPs with pudendal and tibial SEPs. In healthy controls, the S2, S3, and S4 dSEP mean latencies (green) are comparable with the pudendal SEP latency (blue). The tibial SEP latency (pink) is significantly greater than the rest of the EPs. dSEPs, dermatomal somatosensory evoked potentials; SEPs, somatosensory evoked potentials.

In this study, most volunteers were women (*n* = 14; 70%); hence, the female pudendal SEP data were compared with the published female pudendal SEP studies with similar sampling sizes. Five published studies were selected to facilitate the *t*-test for the pudendal SEP, and their sample size, mean latencies, and SD are shown in **Supplemental Digital Content 1** (see **Table 3**, http://links.lww.com/JCNP/A355). This study showed a mean latency of 36.9 milliseconds for the pudendal SEP, 1.5 milliseconds less than the average mean latency of 38.4 milliseconds. One sample *t*-test showed an observed latency difference of 1.5 milliseconds, which is not statistically significant. The comparative study showed that pudendal SEP latencies were comparable with those of S2, S3, and S4 dSEPs. These findings suggest female pudendal SEP latencies can be compared with sacral dSEPs of any sex group but not with the tibial SEP, as shown in Fig. 3 and **Supplemental Digital Content 1** (see **Table 4**, http://links.lww.com/JCNP/A355).

## DISCUSSION

**FIG. 4.**
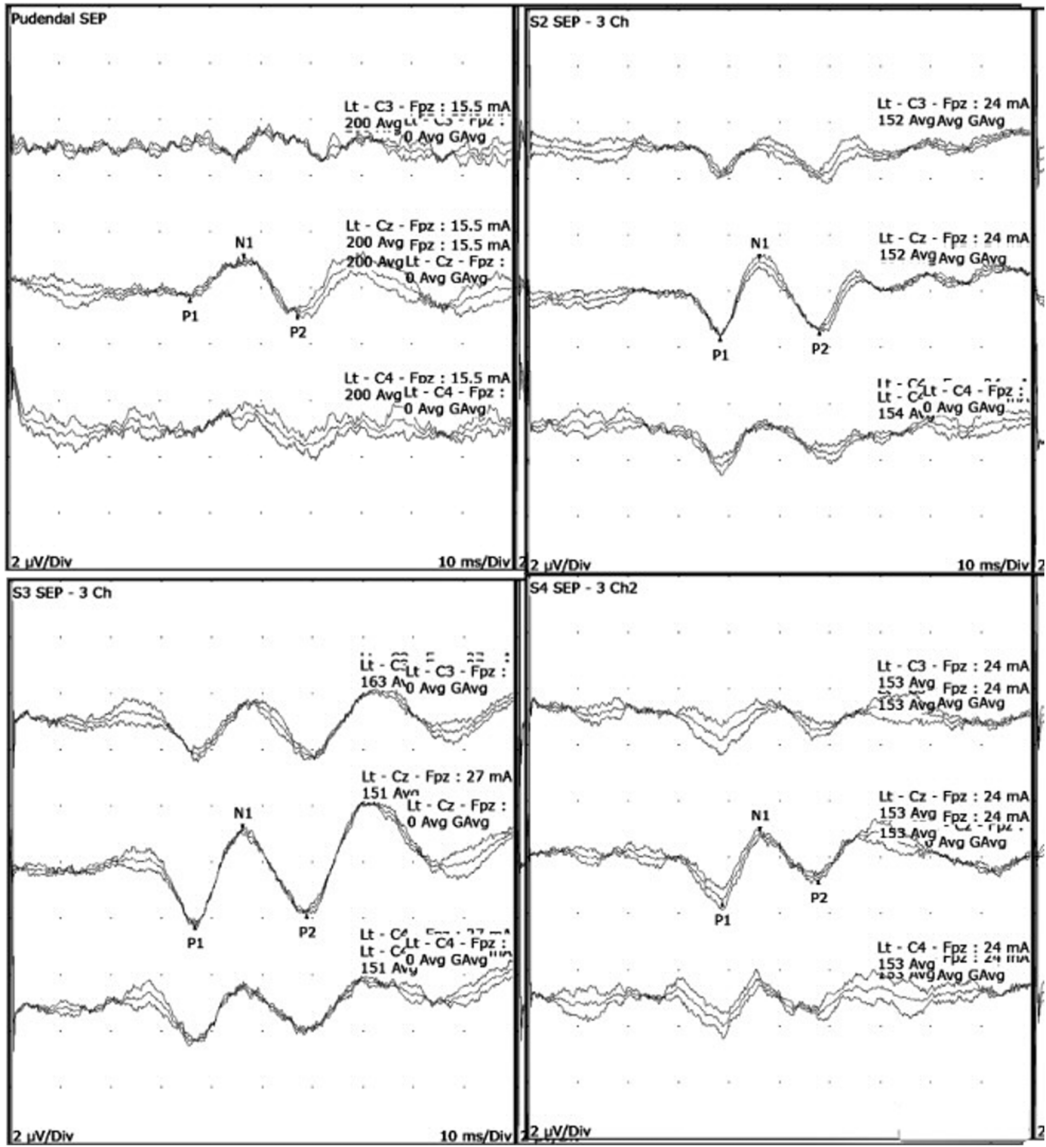
Typical morphologies of pudendal and dermatomal evoked potentials. The S3 dSEP morphology and the latency are similar to the pudendal SEP. somatosensory evoked potentials; SEP, somatosensory evoked potential.

Dermatomal evoked potentials were recorded in all healthy volunteers from bilateral S2, S3, and S4 dermatomes. All sacral dSEPs were reproducible and well tolerated by all volunteers. The dSEP waveforms' morphology and latencies were similar to those of female pudendal SEPs, as shown in Fig. 4 and Table 1. This study did not record lumbar responses, because the primary focus was on establishing the technical parameters and generating normal values from the cortex. In addition, earlier studies have demonstrated that the spinal responses from dSEPs were small and unreliable.^14^ The linear regression analysis showed that age predicts tibial latency by 12.7%, similar to the observations made by Katifi and Sedgwick^17^, which were 12.4%.

**TABLE 1.** Normative Values for Tibial SEP; Pudendal SEP; and S2, S3, and S4 dSEPs

			Mean	SD	Cutoff Value (Mean + 2SD)
Tibial SEP (*N* = 20)					
Latency (milliseconds)	42.5	3.7	22.9 + (0.105) * (age) + (0.094) * (height) + 7.4		
Latency side-to-side difference			0.9	0.6	2.1
Amplitude side-to-side difference in percentage (%)			22	13	48%
S2 dSEP (*N* = 20)					
Latency (milliseconds)			37.3	2.95	15.9 + (0.049) * (age) + (0.117) * (height) + 5.9
Latency side-to-side difference			1.6	1	3.6
Amplitude side-to-side difference in percentage (%)			23.4	15.8	55%
S3 dSEP (*N* = 20)					
Latency (milliseconds)			37.4	2.7	42.8
Latency side-to-side difference			1.3	1.1	3.5
Amplitude side-to-side difference in percentage (%)			20	15	50%
S4 dSEP (*N* = 20)					
Latency (milliseconds)			37.4	3	42 + (−0.187) * BMI
Latency side-to-side difference			1.6	0.8	3.2
Amplitude side-to-side difference in percentage (%)			22	10	42%
Pudendal SEP (*N* = 14)					
Latency (milliseconds)			36.9	2.5	41.9
Latency side-to-side difference			1.3	0.8	2.9
Amplitude side-to-side difference in percentage (%)			17.3	14.2	45.7%

S3, S4, and pudendal SEPS have similar latencies and are not height dependent.

dSEP, dermatomal somatosensory evoked potential; SEPs, somatosensory evoked potentials.

In **Supplemental Digital Content 1** (see **Table 3**, http://links.lww.com/JCNP/A355), the tibial mean latency for volunteer 1 was 34.7 milliseconds at a height of 150 cm. In contrast, volunteer 14 showed a 48.4 milliseconds latency for a height of 183 cm. They both are of a similar age of 30 years, exemplifying that height influences tibial latency. Volunteers 3 and 18 were the same height of 160 cm but aged 20 and 75 years, respectively. Their tibial SEP latencies were 44.3 and 49.8 milliseconds, respectively, showing that age significantly affects tibial SEP latency. This study showed that a combination of age and height parameters would influence the tibial SEP latency by 19.3%. This study also showed that BMI does not affect tibial latency. This study's findings on tibial SEP amplitudes are congruent with the knowledge that independent parameters would not affect tibial SEP amplitudes.^33–35^ These findings suggest that the tibial SEP parameters obtained using surface stimulating pads were similar to those obtained using handheld stimulators, except that the cortical latencies were slower by 2 to 3 milliseconds.

The linear regression analysis showed that a combination of age and height parameters would influence the S2 dSEP latency by 18%. The S3 and S4 dSEP latency data suggest that the significant height variations do not affect the latency in S3 and S4 dSEPs. The S4 dSEP study showed that only the BMI had a 13.4% effect on the S4 cortical latency, but not the height or age parameters. The pudendal SEP latency did not correlate positively with individual age, height, or BMI parameters. These findings were consistent with the published data.^36^ The average cortical latencies of the S2 and S4 dSEPs were comparable, suggesting that the time required for the stimulus to travel the distance between the S2 and S4 dermatomes is minimal, because these impulses travel at a speed of 50 m/second in the peripheral sensory nerve.^37^ Similarly, the distance between the lateral vagina and the external anal orifice is minimal. Hence, the S4 dSEP and the pudendal SEP latencies should be similar. This study showed no significant difference between the S4 and pudendal SEP latencies. The current criteria for reporting the pudendal SEP as abnormal is the prolongation of cortical latency by >47.8 milliseconds or the latency difference between tibial SEP and the pudendal SEP of >7 milliseconds.^38^

Because the patient's height, age, or BMI does not influence the cortical latency of the S3 dSEP, this study proposes an alternative criterion for identifying abnormal pudendal SEP in women: a prolongation of pudendal SEP latency by >42 milliseconds, together with either an interside pudendal latency difference or a cortical latency difference between pudendal SEP and S3 dSEP >3 milliseconds, could be regarded as abnormal. The normative values for S2, S3, and S4 dSEPs are given in Table 1.

### Limitations of the Study

The current sample size is 20, and the stimulating parameters were established based on one examination. The pudendal SEP criteria for the women in this study were determined using a sample size of 14. The male pudendal SEP data were obtained from a small sample size of 6. Future studies should aim for a larger sample size for both male and female populations. The normative values generated for sacral dSEPs ought to be tested in well-defined groups with known cauda equina syndrome or known Tarlov cysts to evaluate the sensitivity and specificity of these dSEPs.

## CONCLUSIONS

Unlike other established evoked potential studies, the current technique is easier to perform and does not induce discomfort for participants. The sacral S2, S3, and S4 dSEPs are consistently recordable and well tolerated when stimulated with surface electrodes. The Tibial SEP can also be obtained without difficulty using stimulating pads. The latencies of S3 and S4 are unaffected by height or age, while S2 latencies are slightly influenced by a subject's age and height by approximately 18%. Dermatomal SEP amplitudes are not dependent on any external parameters. Sacral dSEP latencies are comparable with pudendal SEP but differ from tibial SEP.

## Supplementary Material
