# Supplementary material for: S2, S3, and S4 Sacral Dermatomal Evoked Potentials: Technical Parameters and Normative Values
Source: J Clin Neurophysiol. 2025 Sep 17;43(4):340–6. doi: 10.1097/WNP.0000000000001206 (PMC13124256; doi:10.1097/WNP.0000000000001206)

**Supplementary Materials**

**Material for on-line publication:**

Supplemental Table1: Tibial SEP data along with independent parameters

| No. | Sex | Ht. | Age | BMI | Pain score | R_Tib_Lat | L_Tib_Lat | Mean | R_Tib_Amp | L_Tib_Amp |
| --- | --- | --- | --- | --- | --- | --- | --- | --- | --- | --- |
| 1 | F | 150 | 30 | 17.5 | 1 | 34.7 | 34.7 | 34.7 | 1.9 | 2.6 |
| 2 | F | 165 | 37 | 33.1 | 1 | 45.8 | 44.5 | 45.2 | 1.5 | 1.9 |
| 3 | F | 160 | 20 | 19.5 | 1 | 43.1 | 45.5 | 44.3 | 2.6 | 2.0 |
| 4 | F | 165 | 22 | 26.4 | 1 | 43.3 | 41.3 | 42.3 | 1.8 | 2.2 |
| 5 | F | 165 | 69 | 33.1 | 1 | 42.8 | 42.7 | 42.8 | 2.4 | 3.4 |
| 6 | F | 170 | 27 | 20.8 | 0 | 38.0 | 39.2 | 38.6 | 2.0 | 1.9 |
| 7 | F | 163 | 24 | 28.2 | 1 | 40.6 | 36.6 | 38.6 | 2.9 | 2.0 |
| 8 | M | 168 | 47 | 31.9 | 1 | 43.1 | 40.2 | 41.7 | 1.0 | 0.6 |
| 9 | M | 175 | 27 | 19.6 | 0 | 39.2 | 41.9 | 40.6 | 1.4 | 1.4 |
| 10 | M | 178 | 49 | 24.4 | 0 | 41.6 | 42.7 | 42.2 | 4.1 | 5.3 |
| 11 | M | 190 | 27 | 19.3 | 0 | 41.3 | 43.9 | 42.6 | 0.5 | 1.0 |
| 12 | F | 160 | 36 | 28.9 | 0 | 39.5 | 42.0 | 40.8 | 2.0 | 1.5 |
| 13 | F | 152 | 42 | 39.9 | 0 | 46.4 | 48.6 | 47.5 | 2.3 | 2.1 |
| 14 | F | 183 | 32 | 19.4 | 1 | 47.0 | 49.7 | 48.4 | 2.2 | 1.5 |
| 15 | M | 168 | 31 | 21.3 | 1 | 43.6 | 43.6 | 43.6 | 2.8 | 3.1 |
| 16 | F | 160 | 49 | 25.0 | 0 | 42.5 | 44.8 | 43.7 | 1.7 | 1.4 |
| 17 | F | 150 | 49 | 29.0 | 0 | 40.2 | 38.0 | 39.1 | 3.0 | 3.3 |
| 18 | F | 160 | 75 | 17.9 | 1 | 50.5 | 49.2 | 49.9 | 1.6 | 1.1 |
| 19 | F | 170 | 40 | 23.8 | 1 | 40.8 | 40.6 | 40.7 | 1.4 | 1.8 |
| 20 | M | 177 | 26 | 30.3 | 1 | 41.3 | 42.7 | 42.0 | 2.5 | 2.0 |

R_Tib_Lat: Right Tibial Latency, L_Tib_Lat: Left Tibial Latency, R_Tib_Amp: Right Tibial amplitude, L_Tib_Amp: Left Tibial amplitude. Pain score 0 No pain, 10: worst possible pain, Ht: Height.

Supplemental Table 2: Ten published tibial SEP studies used to calculate the effective size

| **Study Number** | **Name** | **N** | **Mean latency** | **SD** |
| --- | --- | --- | --- | --- |
| 1 | (Misra and Kalita, 1996) | 32 | 41.1 | 4.6 |
| 2 | (Hakatifi, 1986) | 54 | 39.8 | 2.3 |
| 3 | (Dolu et al., 2004) | 30 | 38.3 | 1.59 |
| 4 | (Shaw and Synek, 1985) | 38 | 38.9 | 2.2 |
| 5 | (Restuccia et al., 2000b) | 35 | 38.0 | 2.7 |
| 6 | (Zhang et al., 2011) | 25 | 41.5 | 6.2 |
| 7 | (Eltantawi et al., 2012) | 20 | 38.6 | 1.9 |
| 8 | (Miura, Sonoo and Shimizu, 2003) | 65 | 37.8 | 2.6 |
| 9 | (Chabot et al., 1985) | 27 | 43.4 | 4 |
| 10 | (Chu, 1986) | 160 | 39.3 | 1.8 |
| *Mean parameters of ten studies* | | | *39.7* | *3* |
| *Mean parameters of the current study* | | *20* | *42.4* | *3.7* |

Supplemental Figure 1: Stimulus strength vs cortical latency of S2, S3 and S4 dSEPs.


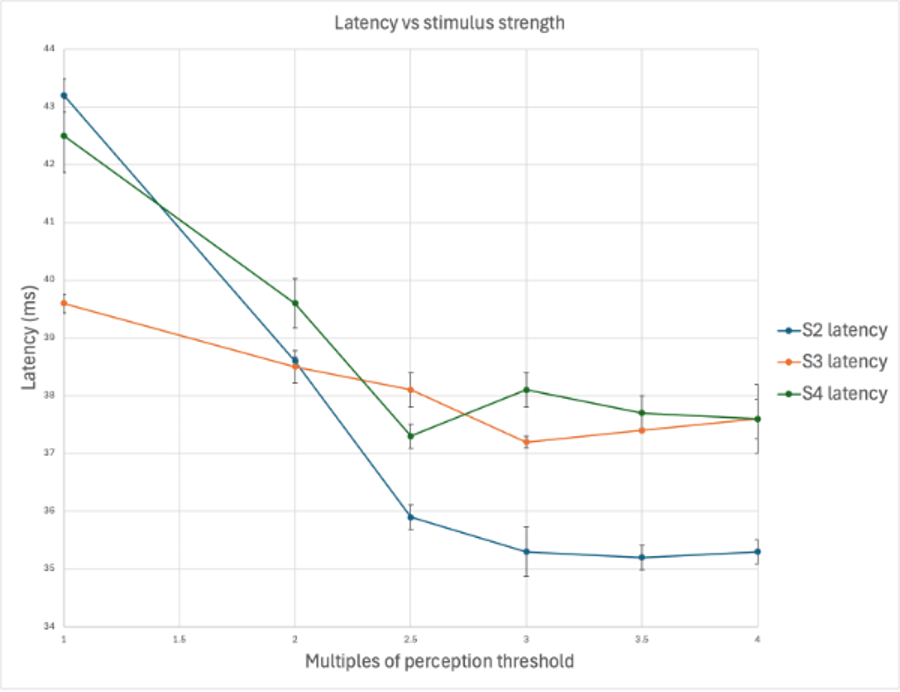


Cortical latencies tend to plateau above three times the current perception threshold in S2, S3, and S4 dSEPs.

Supplemental Figure 2: The impact of the stimulus strength on the dermatomal amplitudes.


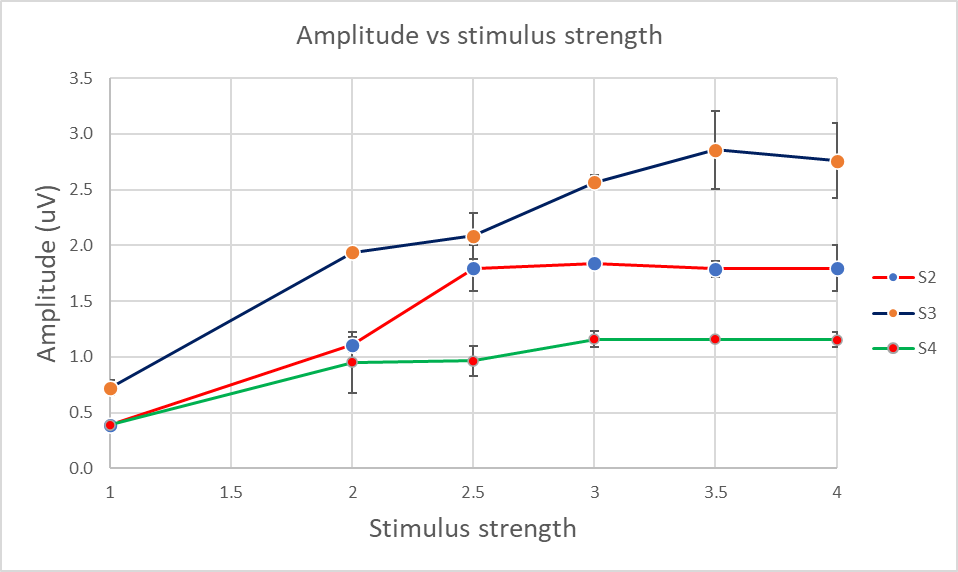


Cortical amplitudes tend to plateau above three times the current perception threshold in S2, S3, and S4 dSEPs.

Supplemental Table 3: Published data for the PSEPs in healthy female subjects.

| Authors | N | Latency | SD |
| --- | --- | --- | --- |
| (Geraldo A. Cavalcanti and Giuliano, 2007) | 11 | 35.7 | 2.4 |
| (Pelliccioni et al., 2014) | 44 | 36.4 | 3.2 |
| (Opsomer et al., 1986) | 10 | 39.9 | 1.63 |
| (Vodusek, 1996) | 12 | 40.2 | 2.2 |
| (Haldeman et al., 1982) | 7 | 39.8 | 1.3 |
| *Published mean* |  | *38.4* | *2.1* |
| *Current study* | *14* | *36.9* | *2.5* |

Supplemental Table 4: Comparative study of Pudendal SEP (N=14) with the entire study 1 group (N=20)

| Parameters | Tibial SEP  (n = 20) ±SD | S2 SEP (n =20) ±SD | S3 SEP  (n =20) ±SD | S4 SEP  (n =20) ±SD | Pudendal SEP  (n =14) ±SD |
| --- | --- | --- | --- | --- | --- |
| Latency | 42.4±3.6 | 37.1±3.1 | 37.4±2.7 | 37.4±3 | 36.9±2.5 |
| Amplitude | 2.1±0.9 | 1.1±0.5 | 1.2±0.7 | 1±0.5 | 1.7±1.2 |
| Threshold | 11±2 | 9.8±3.3 | 10.7±3.1 | 9.7±2.2 | 7.5±1.5 |

S2, S3 and S4 dSEP mean latencies were comparable with the pudendal SEP.

**Material not for on-line publication:**

Permission from 3D4Medical to use their software to generate image of the pelvis.


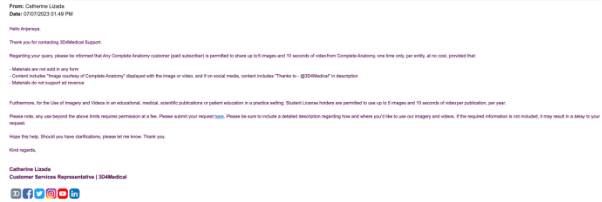

Supplement: Supplementary file 1 [file jcnp-43-340-s001.docx]
